# Effects of Compression Garments on Muscle Oxygen Saturation Recovery in the Upper Limbs Using Near-Infrared Spectroscopy

**DOI:** 10.3390/jfmk10030317

**Published:** 2025-08-15

**Authors:** Maria Teresa Benincasa, Francesco Coiro, Silvia Coppola, Enrico Serra, Ester Celentano, Claudia Costa, Daniele Albano, Rodolfo Vastola

**Affiliations:** 1 Department Neuroscience, Biomedicine and Movement Sciences, University of Verona, 37124 Verona, Italy; mariateresa.benincasa@univr.it (M.T.B.); francesco.coiro@univr.it (F.C.); enrico.serra@univr.it (E.S.); claudia.costa@univr.it (C.C.); 2 Department of Political and Social Studies, University of Salerno, 84084 Salerno, Italy; e.celentano4@studenti.unisa.it (E.C.); rvastola@unisa.it (R.V.); 3 Department of Translational Biomedicine and Neuroscience, Università of Bari, 70121 Bari, Italy; 4 Department of Human, Philosophical and Educational Sciences, University of Salerno, 84084 Salerno, Italy; dalbano@unisa.it

**Keywords:** biceps brachii, compression garments, isometric contraction, post-exercise recovery, sportswear

## Abstract

Background: In recent years, the use of compression garments has expanded into sports contexts to enhance performance and optimize post-exercise recovery. One of the most investigated physiological variables for evaluating their effectiveness has been peripheral muscle oxygenation, a crucial indicator of physical performance. However, studies regarding the effects of compression on the upper limbs remain limited and the topic is insufficiently explored. Therefore, the aim of this study was to analyze the effects of compression garments on muscle oxygen saturation (SmO_2_) recovery in the biceps brachii after brief maximal isometric contractions. Specifically, physiological responses were compared between two conditions (with and without compression garments), hypothesizing that compression would promote faster and more efficient muscle reoxygenation compared to traditional clothing. Methods: Fourteen male participants (mean age: 24.4 years; mean height: 176.75 cm; mean body mass: 73 kg) performed three 10 s isometric contractions separated by 180 s passive recovery periods under compression (CG) and non-compression (noCG) conditions. SmO_2_ was monitored using near-infrared spectroscopy (NIRS), assessing Half-Recovery Time (HRT), Overshoot Amplitude, Initial Slope, and the time constant τ. Results: The compression garment significantly reduced HRT (CG 8.52 s vs. noCG 10.21 s; *p* = 0.035), significantly increased Overshoot Amplitude (CG 21.40% vs. noCG 7.92%; *p* = 0.0014), resulted in a greater Initial Slope (CG 2.43%/s vs. noCG 2.09%/s; *p* = 0.027), and significantly reduced the time constant τ (CG 11.68 s vs. noCG 21.04 s; *p* < 0.001). Conclusions: The use of compression garments demonstrated significant improvements in post-exercise muscle oxygen saturation, suggesting potential advantages for muscle recovery and positive implications for athletic performance.

## 1. Introduction

Compression garments are clothing items designed to exert controlled mechanical pressure on body tissues, with pressure levels varying according to material characteristics and garment fit [1]. Historically employed in medical settings to improve venous return, facilitate lymphatic circulation, and reduce edema [2,3,4,5], these garments have been extensively studied and clinically applied [6,7,8,9,10]. In recent years, the use of compression garments has progressively expanded into sports contexts, aiming to enhance performance, optimize post-exercise recovery [11,12,13,14,15,16,17,18,19,20,21], and reduce perceived exertion during intense physical activities [22]. However, scientific evidence supporting their effectiveness remains controversial. Some meta-analyses and review studies have reported positive effects on recovery, including reductions in creatine kinase, delayed-onset muscle soreness (DOMS), and blood lactate levels [23,24,25,26,27,28]. Conversely, other studies have found no significant differences in performance or physiological responses compared to traditional sportswear [29,30,31]. Peripheral muscle oxygenation is among the most studied physiological variables related to compression, considered a key indicator of physical performance. During exercise, the balance between oxygen availability and muscular demand is crucial to delay fatigue onset [32], making local SmO_2_ an especially valuable proxy for this balance. The principal metrics used to prescribe exercise intensity and monitor training adaptations are heart rate (HR), oxygen uptake (VO_2_), and blood lactate concentration.

Although lactate is a robust marker, its assessment requires intermittent capillary sampling, limiting routine use. HR is easy to obtain but becomes unreliable during resistance exercise and is influenced by numerous confounders such as emotional state, hydration, ambient temperature, humidity, time of day, caffeine intake, and altitude [33]. Moreover, systemic HR and VO_2_ responses exhibit a temporal lag relative to rapid fluctuations in workload [34] whereas local muscle deoxygenation adapts more swiftly under controlled laboratory conditions [35]. Field investigations further indicate that muscle oxygen saturation (SmO_2_) measured with near-infrared spectroscopy (NIRS) can detect changes in muscle activation and metabolic status more sensitively than traditional physiological variables [36,37].

The kinetics of post-exercise muscle reoxygenation provide an integrated window onto the underlying physiological mechanisms and constitute a non-invasive marker of skeletal-muscle oxidative capacity. Rapid resaturation signifies enhanced mitochondrial function, whereas a prolonged return to baseline indicates reduced oxidative capacity [38,39]. The recovery kinetics of SmO_2_, measured with NIRS technology, have been correlated with those of phosphocreatine (PCr) measured by 31P magnetic resonance spectroscopy (31P-MRS) after short bouts of exercise [40], indicating that SmO_2_ reflects muscular oxidative metabolism and the mitochondrial capacity to generate ATP in the presence of oxygen [41,42]. Because PCr resynthesis depends directly on the mitochondria’s ability to generate ATP in the presence of oxygen, the speed of this recovery is regarded as a key determinant in restoring neuromuscular performance after maximal or repeated efforts [43,44]. Accordingly, a rapid resaturation of SmO_2_ reflects superior muscular metabolic capacity, promoting a more effective and faster neuromuscular recovery [45]. The kinetics of SmO_2_ measured by NIRS capture not only oxidative metabolism but also the microcirculation’s ability to swiftly re-establish local perfusion after an ischemic stimulus or intense exercise. Specifically, a rapid resaturation points to superior microvascular function and reactivity, whereas a slow recovery is linked to a transient impairment of the local vascular response [46]. Taken together, these findings establish NIRS-derived SmO_2_ as an integrated biomarker that simultaneously captures microvascular regulation, mitochondrial oxidative metabolism, and neuromuscular recovery capacity [47].

Most research in this area has focused on the long-term effects (beyond 5 min) of compression garments, predominantly examining the lower limbs [26,48].

The upper limbs differ significantly from the lower limbs in anatomy and blood flow characteristics. Arm muscles have a much smaller cross-sectional area and muscle mass compared to leg muscles, and their vascular network is less dense [49]. The vascular anatomy of the upper limb is different from the lower limb, affecting how pressure is distributed and how fluids circulate under compression [50]. Vascular responses differ significantly between upper and lower limbs, suggesting that physiological findings observed in one limb cannot be generalized to the other [51].

To date, only two randomized studies have investigated graduated compression garments for the upper limbs, involving continuous grip activities in climbing and competitive gaming, both showing increased SmO_2_ during recovery [52,53]. Thanks to advancements in non-invasive technologies, such as near-infrared spectroscopy (NIRS), it is now possible to monitor muscle oxygen saturation (SmO_2_), expressed as a percentage, in real time during various types of exercise [54,55,56,57,58]. Specifically, analyzing SmO_2_ values during isometric contractions allows investigation of the relationship between metabolic demand and muscle oxygen availability [59].

During an isometric contraction, blood flow is mechanically restricted; consequently, the rate at which SmO_2_ declines mirrors how quickly the active fibers consume the limited available oxygen. It has been shown that both the slope and magnitude of SmO_2_ desaturation in the brachialis systematically increase with contraction intensity and are reproducible across sessions, confirming that SmO_2_ kinetics provide a reliable, non-invasive index of muscular metabolic demand during submaximal and maximal isometric contractions [60].

This study aimed to analyze the effects of compression garment use on muscle oxygen saturation (SmO_2_) recovery in the biceps brachii following brief maximal isometric contractions. In particular, physiological responses post-exercise were compared between two conditions (with and without compression garments). It was hypothesized that compression garments would promote faster and more efficient post-exercise muscle reoxygenation, thereby enhancing oxidative recovery compared to traditional clothing.

## 2. Materials and Methods

### 2.1. Participants

The sample consisted of 14 male adults, with a mean age of 24.4 years (±2.68), a mean height of 176.75 cm (±6.96), a body mass of 73 kg (±7.34), and an average biceps brachii circumference of 30.5 cm (±2.6). Thirteen participants were right-arm-dominant, and one participant was left-arm-dominant. Participants were recruited based on specific inclusion criteria: regular participation in sports activities (at least three weekly sessions at moderate intensity, totaling at least 5 h per week), and having a biceps brachii circumference between 25 and 35 cm. Subjects with a positive medical history or reported cardiovascular diseases, injuries, or physical conditions potentially influencing test performance, as well as those taking medications capable of altering performance or physiological responses, were excluded from the sample.

### 2.2. Procedures

Participants were instructed to abstain from alcohol consumption and intense training, and to maintain their usual dietary and consumption habits during the 24 h prior to each experimental session. All subjects were informed about the study’s purpose and potential risks and provided written informed consent. The study was conducted in accordance with the principles outlined in the Declaration of Helsinki and was approved by the Ethical Committee of the Department of Human, Philosophical, and Educational Sciences at the University of Salerno (Protocol Number: 0186309). The entire experimental protocol was designed and supervised by the staff of the Laboratory for Innovative Teaching and Sports Performance Analysis at the University of Salerno (Unisa). The tests were conducted in the gymnasium of the University Sports Center (CUS), under controlled environmental conditions (temperature: 22 ± 2 °C; relative humidity: 60 ± 2%) and between 9:00 a.m. and 2:00 p.m. Each participant performed the different experimental conditions 48 h apart.

**Figure 1:**
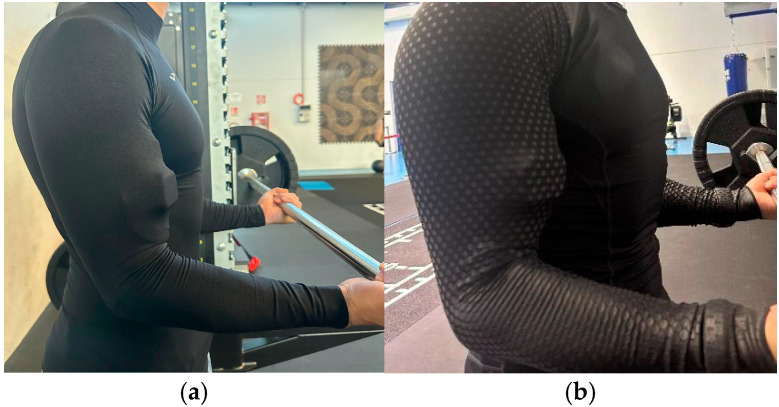
Experimental setup noCG and CG. (**a**) Performance of maximal isometric contraction of the biceps brachii in the noCG condition. (**b**) Performance of maximal isometric contraction of the biceps brachii in the CG condition.

A crossover experimental design was adopted with two conditions (Compression Garment, CG; Control, noCG), separated by a 48 h wash-out period. The order of conditions was randomized using balanced blocks (1:1 ratio). Therefore, each participant took part in two experimental sessions. In the first condition (noCG), exercises were performed while wearing traditional sportswear without compression effects (Figure 1a), whereas in the second condition (CG), the same protocol was carried out using a graduated compression garment provided by LB9 (^®^LB9 BRAND S.R.L. Founded & Endorsed) (Figure 1b). In the noCG condition, participants wore a placebo (“sham”) garment similar in fabric, color, and general appearance to the compression garment but without significant compression. Subjects were not informed about the actual difference between the two garments, thus limiting potential expectancy effects. The measurement of arm circumference was performed with the subject standing upright with the arm relaxed at the side, at the midpoint between the acromion and olecranon processes [61].

Before each session, participants performed a standardized warm-up consisting of three sets of unilateral bicep curls with six repetitions at 50% of 1 RM, separated by one minute of rest between sets, according to the protocol described by Zhao, Nishioka, and Okada [62]. The 1-RM value had been established 48 h earlier in a separate familiarization session following the dynamic strength-testing guidelines of the American College of Sports Medicine [63].

This warm-up, in accordance with ACSM guidelines, increases blood flow and muscle temperature while replicating the movement that will later be held isometrically. Employing a submaximal load during the dynamic warm-up is also consistent with the standard procedure in studies assessing isometric strength, where the dynamic warm-up serves as the baseline condition [64]. During each experimental condition, participants stood upright in front of a barbell secured within a squat rack. The bar was loaded with 120 kg, rendering it immovable and ensuring an isometric contraction. The height of the barbell was individually adjusted to allow participants to maintain an elbow flexion angle of 90° throughout each contraction. Participants performed three maximal bilateral isometric bicep curl contractions, each lasting 10 s at 100% of maximum voluntary contraction [60]. Immediately before every MVC, a 3 s verbal countdown was given, followed by the standardized cue “push as hard as you can!”, delivered by the same investigator in all trials to ensure consistent motivation. Elbow flexion was verified with a handheld goniometer (<3° tolerance); trials exceeding this threshold were repeated.

A passive recovery period of 180 s was provided between each contraction, during which participants remained standing upright with their arms relaxed at their sides [65]. Muscle oxygen saturation (SmO_2_) was monitored in real time during each trial using a near-infrared spectroscopy (NIRS) sensor positioned over the biceps brachii muscle.

### 2.3. Materials

To analyze the effects of compression garments on muscle oxygen saturation (SmO_2_), a compression garment (^®^LB9 BRAND S.R.L. Lavagna, Italy) was used. The garment, made from 60% Nylon and 40% Spandex, size M, was designed to apply graduated compression along the upper limb. Specifically, the pressure recorded at the wrist was 24 mmHg, gradually decreasing along the forearm and subsequently increasing at the biceps brachii muscle, reaching 22.7 mmHg. These data were provided by the manufacturer of the compression garment. These values referred to an arm circumference of approximately 30 cm, corresponding to the average measurement within the analyzed sample, to ensure functional and homogeneous garment adaptation to arm morphology. This approach maintained consistent pressure levels among subjects and standardized the compressive effect on the target muscle. The detailed description of the compression garment and fabric characteristics, in accordance with recommendations by MacRae, Cotter, and Laing [1], facilitates replicability and comparison with previous studies, indirectly contributing to standardizing applied pressure during exercise. Non-invasive measurement of muscle oxygen saturation (SmO_2_) was performed using the MOXY^®^ device (Fortiori Design LLC, Hutchinson, MN, USA), based on continuous-wave near-infrared spectroscopy (NIRS). The sensor was applied to the biceps brachii muscle of the dominant side, consistent with the scientific literature highlighting its sensitivity in reflecting oxygen consumption during muscular contractions [66]. Positioning followed SENIAM guidelines [67], as recommended by the sensor manufacturer, aligning the emitter and detectors parallel to muscle fibers. The sensor was secured using adhesive materials provided by the manufacturer. The device operates at four wavelengths (680, 720, 760, and 800 nm) to measure tissue light absorbance, calculating, through an adaptation of the Beer–Lambert law, the ratio of oxygenated hemoglobin and myoglobin to their total concentration [56,58]. This value is multiplied by 100 and expressed as a percentage, providing the SmO_2_ value within a range of 0 to 100% [68,69]. The parameter enables real-time monitoring of skeletal muscle oxidative metabolism during exercise [70,71]. In addition to SmO_2_, the device also measures total hemoglobin concentration (Thb), reflecting changes in local blood volume [72]. The Default sampling frequency is 0.5 Hz, allowing data acquisition at the four wavelengths for 80 consecutive cycles, producing an average output every 2 s [56,73]. The distances between the emitter and detectors are 12.5 mm and 25 mm, respectively, ensuring an estimated penetration depth of approximately 12.5 mm [56,74]. Adipose tissue thickness (ATT) at the biceps brachii was measured using skinfold calipers [75]. Data were saved in the device’s internal memory and subsequently exported via the “Moxy Monitor Settings” application (version 1.5.5) for processing and analysis; default filter, automatically applied by the device manufacturer, was used to reduce signal noise.

### 2.4. Data Analysis

All SmO_2_ values were normalized relative to baseline, defined as the stable value recorded during the rest period following the warm-up [76]. Specifically, the mean of stable values recorded within a one-minute post-warm-up window was calculated. The recovery phase was defined as the 3 min period following the peak desaturation.

To evaluate the reliability and reproducibility of baseline muscle oxygen saturation (SmO_2_) under the two conditions, the percent coefficient of variation (CV %) and the intraclass correlation coefficient (ICC) were computed using a two-way mixed-effects model (single measurement, absolute agreement).

To verify that contraction intensity was genuinely comparable between sessions, the desaturation amplitude (ΔSmO_2_) for each trial was calculated as ΔSmO_2_ = SmO_2_ baseline − SmO_2_ min. This parameter is acknowledged as a reliable indicator of the mechanical intensity produced by the muscle, as shown by [77] and further studies linking its magnitude to metabolic demands and time to exhaustion [70,78,79].

For each participant and each experimental condition (“Compression” and “Control”), the three ΔSmO_2_ values were averaged to obtain a single representative value. Descriptive statistics (mean ± SD) were calculated for these aggregated ΔSmO_2_ scores, and after verifying the normality of paired differences with a Shapiro–Wilk test, the two conditions were compared using a paired *t*-test. For each comparison, we computed (i) the 95% confidence interval of the mean difference with Student's t distribution, and (ii) the effect size as Cohen’s d, interpreted as follows: 0.2 denotes a small effect, 0.5 a moderate effect, and 0.8 or greater a large effect.

**Figure 2:**
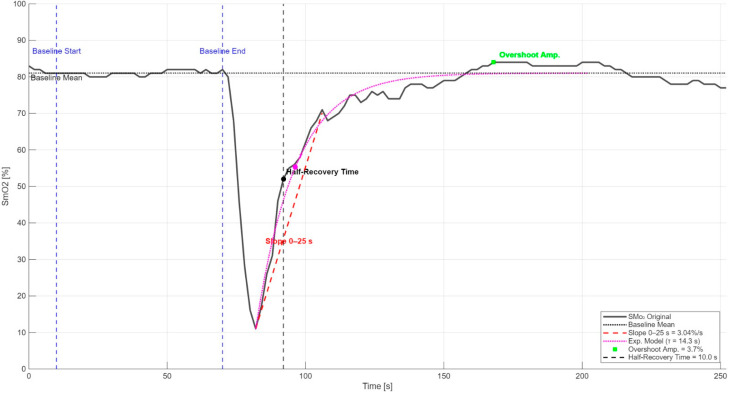
SmO_2_ values and derived recovery parameters. The temporal profile of SmO_2_ in a representative participant is shown by the solid black line. The two blue dashed vertical lines delimit the 60 s baseline window, whose mean is indicated by the black dashed horizontal line. Following the desaturation peak, the black dashed vertical line marks the HRT, when SmO_2_ has recovered half of the difference between its minimum and the baseline. The red dashed line represents the 0–25 s slope (initial re-oxygenation rate), the purple dashed curve with marker depicts the exponential time constant τ from curve fitting, and the green square indicates the Overshoot Amplitude, the peak SmO_2_ exceeding the baseline.

To analyze muscle oxygen saturation (SmO_2_) trends during the post-maximal isometric contraction recovery phase, five distinct metrics were applied (Figure 2).

First, the Half-Recovery Time (HRT) was calculated, defined as the time required for SmO_2_ to rise from the peak desaturation to 50% of the total amplitude between that point and baseline [80,81,82]. Subsequently, the initial recovery slope was analyzed, calculated using ordinary least squares (OLS) linear regression applied to the first 25 s of post-contraction resaturation [83,84]. The time constant (τ) was estimated by fitting a mono-exponential model to the resaturation curve. This parameter describes the overall recovery speed [85] and reflects muscle oxidative capacity [86]. Additionally, the Overshoot Amplitude was determined, defined as the difference between the maximum SmO_2_ peak reached post-contraction and the baseline pre-exercise level [82,87]. Parameters were calculated separately for each participant, obtaining a single representative average value for each of the two experimental conditions. For each subject, the individual difference (Δ) between the means of the two conditions was calculated. The Shapiro–Wilk test was used to verify that differences between experimental conditions met the assumption of normality, necessary for using parametric tests. For parameters meeting the normality assumption (*p* > 0.05 on the Shapiro–Wilk test), the paired Student’s *t*-test was used, while the Wilcoxon signed-rank test was employed for parameters that did not meet this assumption, to compare the two conditions. The magnitude of effects observed with parametric tests was quantified using Cohen’s d index. For the Wilcoxon test, the effect size index r (matched-pairs rank biserial correlation) was reported, with reference values of 0.1 for a small effect, 0.3 for a moderate effect, and 0.5 for a large effect. Finally, to ensure precise and robust evaluation of results, the mean difference (Δ) between the two conditions (No compression–Compression) was calculated. For variables exhibiting a normal distribution, the mean difference and the corresponding 95% confidence interval (95% CI) were calculated using the parametric method based on the paired *t*-test. For variables not meeting the normality assumption, the median difference and 95% confidence interval were calculated using the exact Hodges–Lehmann method, consistent with the non-parametric Wilcoxon test. Additionally, the 95% confidence interval for the effect size indices (Cohen’s d or r) was calculated. Specifically, for calculating the confidence interval of index r, bootstrap resampling with 5000 iterations was employed, providing a precise and robust estimate of the non-parametric effect size, given the non-normal distribution of the analyzed variable. This analysis clearly highlighted the precision of observed effect estimates and thoroughly evaluated individual variability in response to compression. All statistical results were considered significant at *p*-values lower than 0.05.

Fourteen participants were recruited without an a priori power analysis. To examine the robustness of the differences detected in muscle-oxygenation recovery parameters between the two experimental conditions, a post hoc sensitivity analysis was conducted. The analysis indicated that, with the available sample, the smallest detectable effect size was Cohen’s d = 0.49 (equivalent to r = 0.55) at 80% power with a significance level of α = 0.05.

All analyses were performed using MATLAB-R2025a (Version 25.1, MathWorks Inc., Natick, MA, USA).

## 3. Results

The measurement of the athlete’s skinfold thickness revealed a value below 12 mm, the accepted limit for using the NIRS device [68], confirming the validity of obtained data and reliability of muscle oxygen saturation (SmO_2_) measurements.

Statistical analysis demonstrated excellent test–retest reliability of baseline SmO_2_ values between the two conditions, with ICC(3,1) = 0.924. Intra-subject variability, expressed as the percent coefficient of variation (CV %), was minimal (mean = 3.10 ± 3.19%; range = 0.09–11.20%), indicating strong stability of baseline measurements.

ΔSmO_2_ was 77.8 ± 16.9% in the Compression condition and 76.9 ± 17.1% in the Control condition, yielding a mean difference of −0.97 ± 4.72% (95% CI: −3.69 to 1.75%). Paired differences met the normality assumption (Shapiro–Wilk *p* = 0.076); consequently, a paired *t*-test was applied and revealed no statistically significant effect (t_13_ = −0.77; *p* = 0.454; Cohen’s d = 0.21).

**Figure 3:**
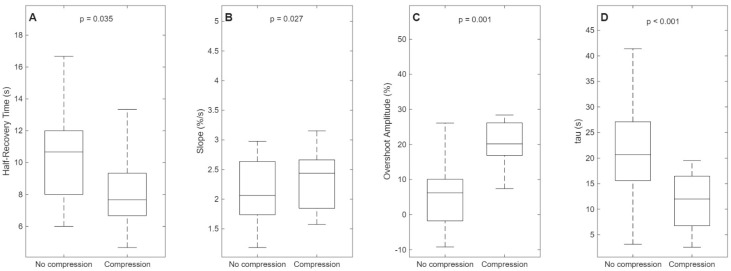
Comparison of muscle oxygen saturation (SmO_2_) recovery parameters between No compression (noCG) and Compression (CG) conditions. (**A**) Half-Recovery Time, defined as the time required to reach 50% recovery of SmO_2_ starting from the minimum desaturation value; (**B**) Initial Slope, corresponding to the initial slope of the resaturation curve within the first 25 s; (**C**) Overshoot Amplitude, indicating the maximum reoxygenation amplitude relative to the pre-exercise baseline; and (**D**) τ, the time constant of the mono-exponential model, indicative of muscular oxidative capacity. The black line within each box represents the median value. The lower and upper edges of the boxes correspond to the 25th and 75th percentiles (interquartile range), respectively, while the dashed vertical lines (whiskers) extend to the minimum and maximum values.

The comparison of Half-Recovery Time between the Compression condition (8.52 ± 2.89 s) and the No compression Control condition (10.21 ± 2.85 s) showed a mean difference of +1.69 ± 2.68 s (No compression–Compression), with a 95% confidence interval between +0.14 s and +3.24 s. This result robustly highlights an improved muscle recovery facilitated by compression garment use. The distribution of differences confirmed normality via the Shapiro–Wilk test (*p* = 0.182). Consequently, a paired *t*-test was applied, showing a statistically significant difference between conditions (*p* = 0.035) (Figure 3A). The effect size, measured by Cohen’s d, was moderate (d = 0.63), with a 95% confidence interval ranging from 0.05 to 1.21, indicating individual variability in muscular response to compression.

The initial recovery slope of muscle oxygen saturation (SmO_2_) under the Compression condition (2.43 ± 0.73%/s) compared to the No compression condition (2.09 ± 0.57%/s) demonstrated a mean difference of −0.34 ± 0.51%/s (No compression–Compression), with a 95% confidence interval between −0.64 and −0.05%/s, indicating a significant increase in the initial recovery speed favored by the compression garment. Again, the distribution confirmed normality (*p* = 0.207) and the paired *t*-test revealed a statistically significant difference (*p* = 0.027) (Figure 3B). The effect size, measured by Cohen’s d, was moderate (d = 0.67), with a 95% confidence interval ranging from 0.09 to 1.24.

Overshoot Amplitude under the Compression condition (21.40 ± 10.91%) compared to the No compression condition (7.92 ± 13.49%) showed a mean difference of −13.48 ± 12.50% (No compression–Compression), with a 95% confidence interval between −20.70% and −6.27%, robustly confirming the positive effect of the compression garment in increasing peak muscle oxygen saturation. This difference was normally distributed (*p* = 0.058). The paired *t*-test indicated a statistically significant difference between conditions (*p* = 0.0014) (Figure 3C). The effect size, assessed via Cohen’s d, was large (d = 1.08), with a 95% confidence interval between 0.50 and 1.66.

The time constant τ under the Compression condition (11.68 ± 5.41 s) compared to the No compression condition (21.04 ± 9.96 s) showed differences that did not meet the assumption of normality (*p* = 0.044). Consequently, the Hodges–Lehmann estimator (median of differences) was +6.90 s, with a 95% confidence interval between 4.22 and 11.65 s. A Wilcoxon signed-rank test was applied, showing a statistically significant difference (*p* < 0.001) (Figure 3D). The effect size was expressed via rank-biserial correlation (r = 0.864); the related 95% CI was estimated through bootstrap resampling with 5000 iterations (0.780–0.881), ensuring a robust estimate of precision.

The post hoc sensitivity analysis confirmed that the sample of 14 participants was sufficiently powered to detect effects as small as d = 0.49 (r = 0.55). All primary parameters displayed effect sizes ≥ 0.63 (HRT d = 0.63; Slope d = 0.67; Overshoot d = 1.08; τ r = 0.86), thus exceeding the predetermined detection threshold.

## 4. Discussion

The results obtained in this study show that the application of compression garments during maximal isometric exercises may have significant beneficial effects on several parameters related to muscle oxygen saturation (SmO_2_) recovery.

The high ICC value (0.924) and the low mean percent coefficient of variation (3.10%) confirm the reliability and reproducibility of muscle oxygen saturation (SmO_2_) measurement by NIRS in our experimental protocol. These results are consistent with the literature for similar instruments and protocols, indicating minimal influence of sensor placement and environmental factors on baseline stability across conditions or measurement days.

The small within-subject variability suggests that any changes detected in later experimental conditions are probably due to physiological adaptations rather than random measurement fluctuations.

Desaturation amplitude during contraction (ΔSmO_2_) was essentially unchanged between the Compression-garment condition (77.8 ± 16.9%) and the Control condition (76.9 ± 17.1%), with a mean difference of −0.97 ± 4.72%. The paired *t*-test revealed no significant effect (t_13_ = −0.77; *p* = 0.454), and the effect size was small (d = 0.21). These data indicate that participants performed maximal contractions of equivalent metabolic intensity in both sessions. Indeed, the literature identifies ΔSmO_2_ as a reliable marker of muscular effort, showing reductions proportional to increasing intensity [77] and documented associations with time to exhaustion and metabolic output [70]. Consequently, the absence of ΔSmO_2_ differences supports the hypothesis that the initial mechanical load was comparable and that any variations seen in recovery parameters reflect solely the effect of the compression garment.

The significant reduction in Half-Recovery Time with compression garments (*p* = 0.035; d = 0.63) suggests a faster muscle capability to restore baseline oxygenation levels. Physiologically, this can be attributed to improved efficiency of venous return and local blood flow, facilitating metabolite clearance and energy replenishment [12,88,89].

The significant increase in Overshoot Amplitude (*p* = 0.0014; d = 1.08) observed with compression garments is particularly physiologically relevant, potentially reflecting enhanced peripheral vascular responses [52,89]. This phenomenon indicates a greater capacity of the vascular system to temporarily enhance oxygen availability, potentially improving local metabolic conditions post-exercise [42]. This observation aligns with research findings commonly reporting increased muscle saturation above resting levels after vigorous exercise. Compression use seems to amplify this effect. DiFrancisco-Donoghue et al. [52], for example, documented that after 15 min of recovery with compression sleeves, forearm SmO_2_ increased by about 9% above baseline values, an effect not observed without compression.

The higher initial recovery slope (Initial Slope, 0–25 s) of the SmO_2_ recovery curve with compression (*p* = 0.027; d = 0.67) indicates increased initial speed in SmO_2_ recovery, further supporting the notion that compression may facilitate immediate reperfusion of muscle tissue, offering potential advantages in muscle management and recovery post-intense activity. The close relationship between muscle reoxygenation and factors such as blood flow, metaboreflex response, and capillary dynamics may explain this finding. Specifically, enhanced blood flow and optimal oxidative enzyme activity in muscles are associated with improved post-exercise reoxygenation capability [90,91]. These results are consistent with findings from a previous study conducted on the lower limbs, which indicated a greater recovery slope of SmO_2_ in the quadriceps using compression garments (*t*-test = 2.69; *p* = 0.021) compared to the Control condition, suggesting enhanced O_2_ delivery in the initial recovery seconds [42].

The significantly lower time constant τ observed under compression garment conditions (*p* < 0.001; r = 0.864) further strengthens the interpretation related to faster metabolic recovery, suggesting that compression improves the speed at which muscles regain oxidative equilibrium post-exercise. This aligns with evidence indicating higher τ values correlate with reduced muscle oxidative capacity [86].

Overall, these findings support the strategic use of compression garments in sports contexts, suggesting potential benefits for improving recovery between intense exercise sessions by facilitating more efficient muscle reoxygenation, potentially enhancing performance during physical activities [23,24]. The data from this study align with previous evidence documenting improved muscle reoxygenation following compression garment use during or after exercise [42,52]. However, other studies did not observe significantly different effects compared to conventional sportswear use [92,93,94,95]. Such discrepancies might be attributed to various factors, including sample characteristics, exercise modalities employed, and differing compression intensities [1,13,96,97].

The possible influence of the placebo effect in studies on compression garments should also be considered, as awareness of their potential benefits can shape participants’ expectations and consequently affect outcomes. Since compression garments exert noticeable pressure on the body, conducting blinded experiments with such clothing is challenging [1,17]. However, in the present study, a tight-fitting sports shirt was used as a control to reduce the placebo effect, potentially strengthening the reliability of the obtained results.

### Limitations and Future Perspectives

A primary limitation of the present work is the lack of concurrent performance metrics, such as maximal force output or electromyographic activity, that could have provided fuller context for the SmO_2_ findings.

A further limitation of the present study is the small sample size (n = 14), exclusively composed of male subjects, which restricts the generalizability of the conclusions to the broader population, particularly women, whose muscle tissue, body composition, and physiological responses to compression may differ significantly.

Future studies should therefore incorporate direct performance measures, such as maximal voluntary contraction and EMG recordings, to link muscle oxygenation kinetics with neuromuscular function. They should also include larger and more diverse samples in terms of gender, age, and training levels to enhance the representativeness of findings. Additionally, investigating the effects of varying compression gradients and materials through dose–response analysis will be beneficial for better understanding the interaction between compression intensity and physiological adaptations.

## 5. Conclusions

The use of near-infrared spectroscopy (NIRS) enabled precise, non-invasive measurement of muscle oxygen saturation (SmO_2_), providing insights into the immediate effects of compression garment usage following intense isometric exercises. The results obtained from this study significantly support the initial hypothesis, demonstrating that compression garments may enhance physiological parameters related to muscular oxidative recovery. Specifically, significant reductions in Half-Recovery Time, substantial increases in Overshoot Amplitude, greater initial recovery slope, and decreased time constant τ were observed, collectively suggesting more effective and rapid muscle reoxygenation. These physiological benefits could primarily stem from improved venous return efficiency and enhanced local metabolic response, thereby facilitating accelerated clearance of metabolites produced during intense muscular activity. In conclusion, the data suggest that compression garment usage may promote faster recovery and improved muscle reoxygenation efficiency following isometric contractions in the upper limbs.

## Data Availability

The data are available at the following link: 10.6084/m9.figshare.29382446.
